# 老年肺癌外科治疗中国专家共识（2022版）

**DOI:** 10.3779/j.issn.1009-3419.2023.102.09

**Published:** 2023-02-20

**Authors:** Lung Cancer Specialty Committee of Chinese Elderly Health Care Association, Enhanced Recovery after Surgery Specialty Committee of Tianjin Medical and Health Association

**Keywords:** 老年患者, 肺肿瘤, 外科, 专家共识, Elderly patients, Lung neoplasms, Surgery, Expert consensus

## Abstract

肺癌是我国60岁以上人群发病率及死亡率最高的癌症。随着社会人口老龄化的日趋严重及肺癌发病率的逐年升高，老年肺癌患者的治疗成为当今社会重点关注的问题。由于胸外科手术技术水平的提高以及加速康复外科在胸外科的应用，越来越多的老年患者可以耐受手术，同时得益于人民健康意识的提高和早诊早筛的普及，更多的肺癌在早期即被发现，使手术治疗的可能性得到提高。但考虑到老年患者的器官功能衰退，加之多种合并症、身体衰弱等因素，亟需针对老年人这一特殊群体的外科治疗方案。因此，我们汇聚了国内相关领域的专家，基于国内外相关最新研究结果，经过充分讨论，针对老年肺癌患者，在术前评估、手术策略方式和切除范围的选择、术中麻醉管理、术后管理等多个方面形成共识性意见，以期为临床工作提供参考和指导。

## Abstract (en)

Lung cancer ranked the highest incidence rate and mortality among people over 60 years old in China. With the increasing social population and incidence of lung cancer, the treatment of elderly lung cancer patients has become a major concern. Due to the improvement of surgical techniques and the application of enhanced recovery after surgery in thoracic surgery, more elderly patients could tolerate surgical treatment. At the same time, with the improvement of health awareness and the popularization of early diagnosis and screening, more lung cancer can be detected at the early stage. However, considering the organ dysfunction, various complications, physical weakness and other factors of elderly patients, it is important to provide individualized surgical treatment for the elderly. Therefore, based on the latest research progress worldwide, the experts in relevant fields have developed this consensus which contributes to guide the preoperative evaluation, surgical strategy, intraoperative anesthesia management, and postoperative management for elderly patients of lung cancer.

## 前言

肺癌是我国60岁以上人群发病率及死亡率最高的癌症^[1]^，随着中国社会老龄化程度加重以及肺癌发病率的逐年上升，中国老年肺癌患者的群体基数达到了历年来的高峰。由于人民健康意识的提高和早诊早筛的普及，越来越多的肺癌患者在早期即被发现，手术治疗的可能性得到提高。但是由于老年患者的器官功能衰退，加之多种合并症、身体衰弱等因素，术后并发症发生风险显著增加。因此，老年肺癌患者是否能耐受手术，如何降低围手术期风险、减少并发症、保护术后器官功能状态，成为当今社会重点关注的问题。

当前，随着加速康复外科（enhanced recovery after surgery, ERAS）理念的提出，ERAS在胸外科的应用也越来越普及，尤其是在肺癌患者围手术期的管理方面，理念所倡导的多模式治疗方法和多学科合作已得到认可，尤其对于器官功能状态趋于弱化的老年患者群体有更重要的价值。《中国加速康复外科围手术期管理专家共识》从多学科角度出发，结合ERAS理念对外科手术进行了优化建议。2015年，美国外科医师协会（American College of Surgeons, ACS）与美国老年医学会（American Geriatrics Society, AGS）联合颁布了《老年患者围手术期管理指南》 ^[2]^。美国国立综合癌症网络（National Comprehensive Cancer Network, NCCN）每年会公布新版的《NCCN肿瘤学临床实践指南——老年肿瘤》^[3]^，最新版公布时间为2022年7月，该指南指出，老年肿瘤患者在新的肿瘤疗法的临床试验中的代表人数不足，因此，缺乏循证医学资料来指导老年肿瘤患者的治疗。同时由于老年肿瘤患者具有更多的耐受性/合并症问题，且自然年龄相同或相近的人群的生物学年龄差别较大，因此建议组成包括外科、内科、放疗科、麻醉科、康复科、老年学科、营养科等跨学科团队，进行术前综合评估和围手术期个体化管理^[4]^。2015年，中华医学会老年医学分会与解放军总医院老年医学教研室联合制定了《老年患者术前评估中国专家建议（2015）》^[5]^，就中国老年患者的术前评估方法给予了建议。基于上述的指南、专家共识和专家建议，中国老年保健协会肺癌专业委员会为了更好地达成共识，略去执行复杂的条目，使胸外科医生在面对老年肺癌患者时能更方便地参考和应用，故撰写了本共识。

根据世界卫生组织（World Health Organization, WHO）的相关规定，老年患者的年龄界定可以分为：（1）年龄在65岁-75岁之间的低龄老年患者；（2）年龄在76岁-85岁之间的老年患者；（3）年龄超过85岁的高龄老年患者^[6]^。单凭实足年龄估计预期寿命、身体功能储备或者并发症风险并不完全可靠。当临床医生不能准确预测患者个体的预期寿命时，根据NCCN老年肿瘤患者的诊疗指南和其他文献数据^[7⇓-9]^，对患者与年纪相当的平均水平普通人相比是否有更长生存期做出估计是可能的。本共识对老年肺癌的年龄界定以WHO老年患者的年龄界定为准，以65岁作为老年界限。

年龄并非手术风险的主要考虑因素，但患者的生理状况仍需要进行术前系统评估^[10,11]^。但是，随着年龄的增长，患有早期疾病的老年患者选择不接受外科治疗的可能性会增加，而在真实世界中，患者体力状况和合并症对于是否接受手术治疗的影响远大于年龄^[12]^。因此对于老年患者而言，外科手术治疗的策略及围手术期的高质量管理是决定患者预后的重要因素。因此，专委会建议，由于肺癌的外科治疗通常为创伤性手术而非功能修复性手术，在真实世界中，80岁以下老年肺癌患者的外科治疗策略可能更加宽松，对于80岁以上老年患者的手术选择往往要经过多学科综合会诊评估决定，而85岁以上老年肺癌患者在有替代治疗方法的情况下应更加慎重地选择是否接受外科治疗，除非万不得已的急诊手术，因为手术带来的并发症风险会显著提高。

问题1：老年肺癌患者如何进行术前评估

共识推荐：老年肺癌患者术前需接受全面手术风险评估，确定是否适宜接受手术干预。评估建议包括综合性老年医学评估（comprehensive geriatric assessment, CGA）、美国麻醉医师协会（American Society of Anesthesiologists, ASA）分级、呼吸系统评估、心血管系统评估、内分泌系统评估和营养评估等。

证据: 对于老年肺癌患者是否接受外科治疗的决策不应仅考量近期预后，更要考量患者有无严重并发症或合并症，老年患者手术是否获益主要看远期预后，比如是否可以延长患者健康预期寿命、术后是否能保持或接近患者术前机体功能状态，显著影响术后生活质量的外科治疗不可取。是否接受手术需在客观详实地将相关问题（表1）告知患者或家属后，由医患双方共同决定^[13]^。术后不良结果的高风险因素包括可能危及生命的术前合并症（重要脏器的血栓性疾病和功能障碍）、认知功能损害、躯体功能障碍、营养不良。只有医患双方对手术的目标认知一致，才建议继续进行后续的风险评估及术前管理。患者在胸外科门诊完成上述初步评估后，门诊护士应分发住院前告知书并进行宣教，包括戒烟（至少术前4周）、戒酒（至少术前4周）、改善营养状态及控制感染和血栓评估等。

**表1:** 老年肺癌患者手术决策前问题

序号	老年肺癌患者手术决策前问题
1	患者手术后有无可能丧失大部分肺功能?
2	术后是否可能需要长期住院或他人长期照料? 为此，医院、患者及家属是否有准备?
3	如果放弃外科治疗，对患者健康的影响有多大?
4	患者是否知晓自己的病情? 自主意愿希望/不希望得到什么样的治疗?
5	如果患者已经知晓病情，本人是否愿意接受手术?
6	手术所能达到的效果是否与患者/患者家属的预期相一致?

老年肺癌患者术前评估的目的是为了确定老年患者是否适宜接受手术干预。老年肺癌患者术前评估建议包括CGA、ASA分级、呼吸系统评估、心血管系统评估、内分泌系统评估、营养评估等。老年患者机体功能低下、合并症多、合并用药多、营养状况差等，这些均是影响患者治疗的客观因素，应尽量避免治疗不足或过度，营养状况较差者不宜接受外科治疗。

（1）CGA

CGA的实用性在老年肿瘤患者中已得到证明，而已有资料^[14,15]^表明CGA（合并症、功能状态、认知功能、老年症候群、多重用药和营养状况）与老年患者的生存有关。但其内容比较复杂，临床实际应用起来并不方便，可能对于全部患者而言不具有操作性，因此部分学者制定了针对老年肿瘤患者的改良CGA评估量表（表2）；这种方法可用于估计预期寿命、检测未发现的健康问题、提高预后及患者的依从性。

**表2:** 改良综合性老年医学评估

序号	评估领域	评估内容
1	基本人口特征	年龄、性别、种族、教育程度、生活安排等
2	合并症	采用美国老年资源与服务（Older Americans Resources Study, OARS）合并症评分量表评估32种合并症，根据疾病对日常活动影响程度（不影响、轻微影响和明显影响）评分为0分-96分
3	日常活动评分	采用欧洲癌症研究与治疗组织生活质量调查问卷（European Organization for Research and Treatment of Cancer Quality of Life Questionnaire-Core 30, EORTC QLQ-30），通过5个问题了解在吃饭、穿衣、洗澡、如厕、运动和行走方面的限制，评分为0分-100分
4	功能状态	采用工具性日常生活活动（Instrumental Activity of Daily Living, IADL）评分和运动评估量表两种评估工具，评估独立生活能力和获取帮助的能力（不需要帮助、需要帮助、完全做不了），评分为0分-21分；运动评估量表通过3个问题将患者分为四类：经常剧烈运动、经常长时间行走、经常短时间行走、偶尔运动
5	疼痛评估	评估患者在1周之内的疼痛情况，评分为0分-10分，0分为无痛，10分为最强烈剧痛
6	经济状况	采用老年人资源与服务（Older American Resource and Services, OARS）评分法，从严重财务困难到无财务困难，评分为3分-9分
7	社会支持	采用医疗结局研究（Medical Outcomes Study, MOS）量表，通过20个项目评估患者在4个分量表中（情感支持、有形的支持、深切支持、积极互动）获得社会支持的情况，每个项目分为5级（从没有至随时有），总评分为0分-100分
8	情感状态	采用医院焦虑和抑郁评分量表，共14个评估项目，焦虑和抑郁分量表各有7个评估项目，每个项目分为4级（大部分时间、经常、偶尔、从不），总评分11分以上代表焦虑或抑郁
9	精神福祉	采用信仰量系统来评估精神信仰与实践以及从共享信仰团体得到社会支持的情况，通过15个评估项目、4级评分法，从无信仰到高级精神福祉，评分为0分-45分
10	生活质量	采用EORTC QLQ-30评分，分量表包括疲劳、不适、一般身体症状、身体机能、社会机能、精神困扰，从质量差到质量高总分为0分-100分

（2）麻醉评估

建议采用ASA分级标准对老年肺癌患者机体功能与合并症情况进行术前评估（表3）。I级：围手术期死亡率为0.06%-0.08%；II级为0.27%-0.40%；III级为1.82%-4.30%；IV级为7.80%-23.0%；V级为9.40%-50.7%；超过85岁的高龄老年肺癌患者，一般不适宜接受任何肺癌相关外科治疗，但也存在部分身体状况较好且年龄超过85岁的患者接受了肺癌相关外科手术治疗，这一定需要经过慎重评估受益与风险，如实性肿瘤位于肺外周，可行姑息性创伤小、恢复快的电视辅助胸腔镜下楔形切除术；而年龄介于76岁-85岁的老年肺癌患者，应慎重经由多学科综合评估。若分级为I级-II级，可接受外科治疗；分级为III级及以上，不适宜接受外科治疗。年龄在65岁-75岁的低龄老年患者，外科策略的选择可与正常成年人等同。

**表3:** 美国麻醉医师协会分级标准

分级	器官功能状况
I级	体格健康，发育营养良好，各器官功能正常
II级	除外科疾病外，有轻度合并症，功能代偿健全
III级	病情严重，体力活动受限，但尚能应付日常活动
IV级	病情严重，丧失日常活动能力，经常面临生命威胁
V级	无论手术与否，生命难以维持24 h的濒死患者

（3）呼吸系统评估

肺部手术对于周密的术前呼吸系统评估依赖性高，患有肺部基础疾病或肺功能降低的老年患者术后并发症的发生率明显高于其他年龄段患者，因此，术前呼吸系统评估和准备至关重要。肺功能检查（pulmonary function test, PFT）是最常用的术前肺功能评估方法，可以真实反映患者的通气功能、弥散功能以及是否存在气道阻塞等情况。该检查有助于帮助手术医生评估手术是否可行和术后肺部并发症的发生风险，也有助于选择手术方式和范围（如胸腔镜手术或开胸手术，肺段切除或肺叶切除）。因此，年龄>65岁拟接受外科治疗的老年肺癌患者均需术前行PFT。在PFT的各项指标中，第一秒用力呼气容积（forced expiratory volume in one second, FEV_1_）是预测肺相关手术风险的独立危险因素^[16]^。PFT指标异常，尤其是FEV_1_较低的患者，其术后肺部并发症发生风险较高，术前应充分评估手术风险，并采取相应措施在术前尽量提升肺功能储备，降低术后并发症发生风险。对于合并肺部基础疾病的老年肺癌患者术前建议给予以下治疗：①应用恰当的措施如抗生素控制和治疗急慢性肺部炎症；②对合并支气管哮喘或有明确的支气管痉挛病史的老年患者，建议术前至少1周开始使用吸入性长效糖皮质激素、长效抗胆碱能药物及长效β2受体激动剂，以改善患者术前肺功能、降低术后肺部并发症发生率^[17]^；③使用促排痰措施，如雾化治疗，并指导老年患者进行呼吸锻炼，如呼吸操及使用呼吸训练器械，还应练习深而慢的腹式呼吸，上述措施有助于降低术后肺部并发症的发生率^[18]^。PFT结果异常时，可以进一步行心肺功能运动试验（cardiopulmonary exercise testing, CPET）、爬楼梯试验及6分钟步行试验（6 minute walking test, 6MWT）。6MWT主要用于评价合并中重度心肺基础疾病的老年患者对治疗措施的疗效，评估患者的躯体功能状态，可作为老年患者的重点监测指标之一，也是患者生存率的预测指标之一。术前的爬楼梯试验可以较好地反映术后并发症的发生风险。检测结果较差的患者应接受CPET^[19]^。若CPET检测过程中患者的血氧饱和度（oxygen saturation, SPO_2_）降幅大于15%，建议慎重选择手术治疗^[20]^。

（4） 心血管系统评估

一般情况下通过患者的心脏病史、目前症状、运动耐量可快速了解患者的心血管耐受情况，但必要时冠状动脉CT血管造影（computed tomography angiography, CTA）、超声心动图、心肌核素显像、颈动脉超声及下肢静脉超声等检查仍是患者是否适合手术的重要检查。肺切除术作为高风险手术，必要时可行核素心肌负荷显像等更进一步的心脏影像学检查^[21]^。Goldman心脏风险指数可帮助评估老年肺癌患者围手术期发生心脏不良事件的风险：总分0分-5分患者心源性死亡率为0.2%，危及生命的并发症发生率为0.7%；总分6分-12分患者分别为2%、5%；总分13分-25分的患者分别为2%、11%；总分≥26分的患者则分别为56%、22%。

对于合并心血管系统基础疾病的老年患者，应在术前给予积极的内科治疗，包括：①血压控制稳定；②症状较轻的心力衰竭患者可考虑术前加用血管紧张素转换酶抑制剂类药物；③既往长期服用β受体阻滞剂或他汀类药物的患者，应持续服用；对于合并冠状动脉粥样硬化性心脏病患者，可考虑至少在术前2天加用β受体阻滞剂并在术后持续使用，以达到心率在静息状态下60次/min-70次/min且收缩压>100 mmHg^[21]^。

（5）内分泌系统评估

术前应详细询问患者是否患有糖尿病、肾上腺疾病、甲状腺功能亢进（甲亢）或甲状腺功能减退（甲减）等，若否认相关病史，也应在术前检查中加入相关的系统检查，以明确是否存在患者未知的隐匿性疾病。合并糖尿病的老年肺癌患者有合并多器官功能障碍的风险，糖尿病也被认为是围手术期心脏并发症的中危因素，故术前评估应注重评估是否已存在其他器官损伤及血糖水平是否得到良好控制。

严重的甲亢或甲减都可能会增加老年患者围手术期并发症发生风险。甲亢患者可能合并有心律失常（如心动过速）、震颤或腹泻等。甲减患者可能合并有心动过缓、低血压、体重增加、嗜睡、心包积液、心功能下降、缺氧及高碳酸血症等。老年患者的甲亢、甲减症状和临床表现可能较年轻患者更不明显且无特异性，需在术前功能评估中特别加以关注。若老年肺癌患者因咳血、急性气道梗阻等需行急诊手术时，甲亢患者应在术前使用β受体阻滞剂、抗甲状腺药物及糖皮质激素治疗。对于甲状腺替代治疗丙硫氧嘧啶等抗甲状腺药物，手术当日需按常规用药。

（6）营养状况评估

对于老年肺癌患者，术前的营养状况评估至关重要，可参考营养风险评估（nutritional risk screening 2002, NRS 2002）筛查营养不良风险（表4）。该量表可评估患者营养状况，结果根据患者年龄、体重、饮食情况、疾病损伤状况而定。其中，NRS 2002评分≥3分者存在营养风险，建议暂缓手术，应考虑术前营养支持2周以上再次评估营养状况，<3分者无术前营养风险。

**表4:** 营养风险评估2002

项目	营养状况	疾病状态
0分	营养状况正常	疾病状态稳定
1分	3 个月内体重丢失>5%或近1周摄食量较疾病前减少25%-50%	慢性疾病急性加重、骨折、肿瘤、糖尿病、肝硬化、血液透析、慢性阻塞性肺疾病（chronic obstructive pulmonary disease, COPD）
2分	2个月内体重丢失>5%或身体质量指数（body mass index, BMI）18.5 kg/m^ 2^- 20.5 kg/m^ 2^，加上受损的基本营养状况或近1周摄食量较疾病前减少50%-75%	拟行大手术、中风、严重肺炎
3 分	1个月内体重丢失>5%（或3个月内体重下降15%）或BMI<18.5 kg/m^ 2 ^，加上受损的基本营养状况或近1周摄食量较疾病前减少75%-100%	脑损伤、骨髓移植、重症监护室（intensive care unit, ICU）患者[急性生理学及慢性健康状况评分系统（Acute Physiology and Chronic Health Evaluation Scoring System, APACHE）>10]

总分=营养状况评分+疾病严重程度评分+年龄评分（年龄≥70岁加1分）

问题2：老年肺癌患者接受外科手术策略的考量因素

共识推荐: 手术是老年早期肺癌患者的最有效治疗选择。年龄与合并症状况是老年肺癌患者手术治疗策略需要综合考量的重要指标。

证据：多项研究^[22,23]^证明了手术治疗老年肺癌患者甚至80岁以上老年肺癌患者的可行性。需要注意的是，老年早期非小细胞肺癌患者存在明显与年龄相关的治疗偏倚。然而，近年来一些数据也显示接受手术切除的老年非小细胞肺癌患者病例数在逐渐增加。此外，还应注意伴随着手术率的增加，老年肺癌患者手术后的中位生存率有所改善^[24,25]^。这种潜在的治疗偏倚现象可能与治疗地外科医生的治疗经验及技能程度有关。数据分析^[26]^发现，65岁以上的老年肺癌患者在胸外科平均手术量较低的地区手术率仅不到63%，而在胸外科平均手术量较高的地区手术率高于79%。而尽管一些病例对照研究结果表明老年患者术后死亡率较高，但大型随机试验的数据^[27,28]^其实并不支持这一结论，在住院时间和术后并发症发生率方面，无论年轻还是老年患者，两项研究均未显示出明显差异。当然，年龄也并非对外科治疗全无影响，有研究^[28]^证实，术后并发症的发生率与年龄75岁以上、男性、合并症较多、肿瘤体积较大以及在肺癌相关手术量较少的医疗中心进行治疗有关。即使老年肺癌患者的术后并发症发生率及死亡率略高于年轻患者，但整体发生率仍处于较低水平，因此手术仍是老年早期肺癌患者的一种有效治疗选择。老年肺癌患者的长期生存率与其治疗机构内的手术量呈正相关。尽管手术量大的医疗机构内高危患者更多，但手术量越大的医疗中心肺癌手术相关死亡率更低^[26]^。同时，也有证据^[29]^表明，肺癌手术较差预后的风险并非仅取决于年龄，而是与年龄和疾病分期均有关。分期为I期的老年肺癌患者，不管年龄<75岁还是≥75岁，术后发生严重功能障碍或死亡的风险为低风险（16%）；分期为II期-IIIA期，不伴或仅伴轻度合并症的≥75岁老年肺癌患者，术后发生严重功能障碍或死亡的风险为中风险（33%）；分期为II期-IIIA期且患有中度或重度合并症的≥75岁老年肺癌患者，术后发生严重功能障碍或死亡的风险为高风险（60%）。因此，年龄与合并症状况是术前需要综合考量的指标。

问题3：老年肺癌患者如何选择手术切除范围和术式

共识推荐：肺叶切除术被认为是I期肺癌外科治疗的金标准，但对于老年肺癌患者，出于安全考虑，应在必要时适当缩小切除范围，可采取亚肺叶切除的方式。微创手术是首选方式。

证据: 关于手术切除范围的选择，尽管肺叶切除术仍被认为是I期肺癌外科治疗的金标准，但对于老年肺癌患者，出于安全考虑，应在必要时适当地缩小切除范围，肺段切除或楔形切除患者的长期生存率与行肺叶切除术的患者相当，且患者生活质量更好^[30⇓-32]^。建议尽量避免全肺切除术，该术式与老年人术后并发症发生率和死亡风险升高有关^[33,34]^ 。

关于手术方式的选择，随着胸腔镜技术的进步，毋庸置疑的是，胸腔镜手术与开放手术相比，创伤性更小，住院时间明显缩短，术后并发症更少^[35,36]^，使得无论从主观上还是客观上，早期老年肺癌患者应选择胸腔镜手术而非开放手术。术前应做好手术规划和应急预案，优化手术流程，尽量缩短手术时间。术中应尽量避免过度牵拉、挤压肺组织。如果在术中遇到不得不开放的情况，应尽量维持胸廓的完整性，注意保护重要神经结构，如喉返神经、膈神经及迷走神经等。注意精细操作，减少术后长时间肺漏气的情况发生。应尽量避免大出血和大量输血。

问题4：老年肺癌患者术中麻醉管理的注意事项

共识推荐: 术中麻醉用药要考虑老年肺癌患者的年龄和基础身体状况等个体差异，合理应用麻醉药物，控制术中补液，实施肺保护性通气策略，减少术中肺损伤，降低术后肺部并发症的发生率。

证据:

（1）麻醉用药策略

麻醉用药可对全身产生广泛影响，麻醉过浅会导致术中体位变动、应激反应或代谢异常、术中知晓等情况的发生。麻醉过深会导致呼吸抑制、缺氧、苏醒延迟或免疫抑制等并发症的发生。理想的麻醉方法和药物选择原则是：术中镇静、止痛和肌松作用好；手术不良反射阻断满意；术后苏醒恢复快。考虑到老年患者个体差异大，麻醉药物的选择应综合考虑患者身体情况、手术方式、手术时长等因素，由麻醉科、外科医生共同商议决定。优先选用短效的麻醉药物用于全麻诱导和全麻维持，例如丙泊酚、芬太尼或舒芬太尼、瑞芬太尼、罗库溴铵等。为应对肺癌手术引起的呼吸循环紊乱，胸外科多采用全身麻醉并使用双腔气管插管，但随着快速康复以及“无管化”手术理念的发展，无气管插管和不留置导尿管下的胸腔镜手术也逐渐成为一些医学中心的选择。

（2）术中输液策略

限制性输液策略会引起组织灌注不足及脏器功能衰竭；开放性输液策略会造成循环液体过量、肺等组织水肿或心脏超负荷等。对于老年患者而言，术中术后更易发生心功能不全和肺水肿，术中应保证静脉通路通畅，限制补液总量并控制输液速度^[37]^，基于患者年龄和基础身体状况的术中目标导向液体治疗是减少围手术期心功能不全导致的心血管意外以及肺损伤的有效方法^[38]^。

（3）机械通气以及肺保护性通气策略

肺癌手术大部分情况下需要使用双腔气管插管并在术中使用单肺通气。手术中不适当的单肺通气策略会造成术中呼吸机相关的肺损伤，增加术后肺部并发症的发生率。有证据^[39]^显示，术中使用保护性肺通气策略可以减轻肺损伤发生风险，降低术后并发症发生率并加速术后康复。对于老年患者而言，可通过以下几种通气方式来实施肺保护性通气：①小潮气量通气（6 mL/kg-8 mL/kg的潮气量通气，可减轻气道压力及剪切力所导致的机械损伤和肺部炎性反应）；②通气侧使用呼气末正压通气（positive end expiratory pressure, PEEP）：使用适当的PEEP（6 cm H_2_O-8 cm H_2_O）可以促进肺泡复张，减少剪切力，维持正常的通气血流比；③肺复张策略：对老年机械通气患者可每隔30 min-45 min实施一次手法肺复张，目前比较推荐采用PEEP递进法来代替手动肺复张法^[40]^。此外，术中应注意控制吸入氧气浓度（fraction of inspiration O_2_, FiO_2_）在30%-50%，同时保持SPO_2_不低于94%。高FiO_2_仅在术中突发低氧血症时做紧急处理。

问题5: 老年肺癌患者术后管理的注意事项

共识推荐: 术后采用多模式镇痛策略，促进老年患者早期的膈肌运动和咳嗽排痰，但避免镇静药物的使用；术后尽早恢复下肢运动锻炼、术后肺功能康复呼吸训练。

证据：

（1）合理镇痛

术后合理镇痛可促进老年患者早期的膈肌运动及咳嗽排痰，进而加速肺功能的恢复并减少术后肺感染的发生。术后镇痛常用药物及方法如下：①阿片类药物；②非甾体类抗炎药；③局部麻醉药；④肋间神经阻滞。多模式镇痛策略包括阿片类药物与局部麻醉药复合用于区域阻滞、局部麻醉药切口浸润（区域阻滞或神经干阻滞） 联合全身性镇痛，使患者早期活动、减轻围手术期应激反应。建议个体化评估老年肺癌患者的术后疼痛情况，将术后镇痛效果最大化，同时避免过度镇静导致呼吸抑制。此外，选用口径较细的胸腔引流管并在术后尽早拔除可减轻患者术后疼痛。可使用老年肺癌患者术后镇痛记录及监测表评估老年患者术后疼痛情况（表5）。

**表5:** 老年肺癌患者术后镇痛记录及监测表

项目	老年肺癌患者术后镇痛
镇痛药物记录	
	药品通用名、剂量、使用方法
	静脉镇痛泵参数设置：背景输注剂量、单次额外注射剂量、维持时间
	有效药物剂量的总和
	突破痛后额外补充镇痛药物的剂量
监测项目记录	
	疼痛评分、缓解时间
	是否出现低血压、心动过缓或心动过速
	呼吸频率和动度
	胃肠道反应、恶心呕吐、食欲不振
	皮肤瘙痒、皮疹
	运动阻滞、感觉水平的下降

（2）尽早下地活动

术后早期恢复运动锻炼对于预防下肢静脉血栓形成及降低肺栓塞风险具有非常重要的意义^[41]^，同时是预防术后肺部感染的重要手段^[42]^。术后第1天患者尽早下地活动可以增加肺部通气量，促进伤口愈合，降低术后肺部并发症的发生率，并能显著缩短住院时间^[43]^。

（3）术后谵妄和认知功能障碍

老年患者因为身体机能、药物敏感度与药物清除能力的降低，导致肺癌术后谵妄的发生率增加。减少术后谵妄的措施包括：术中避免低氧状态和维持血流灌注，维持血清钠、钾正常，控制血糖，减量或停用抗组胺药、苯二氮䓬类药物及哌替啶。术中及术后应用咪达唑仑镇静是术后谵妄的独立危险因素，应尽量避免使用^[44]^。

术 后 认 知 功 能 障 碍（postoperative cognitive dysfunction, POCD）是一种术后人格、社交能力及认知能力和技巧的变化，其危险因素包括年龄、麻醉时间过长、呼吸系统并发症、术后合并感染、非计划二次手术、受教育水平较低等。POCD是发生术后并发症、住院时间延长以及术后6个月内总死亡率增高的风险因素^[45,46]^。目前，老年POCD尚缺乏有效的治疗方法，提高麻醉管理水平及围手术期呼吸、循环系统管理水平，纠正老年肺癌患者术前合并症，围手术期合理用药，是预防术后POCD的有效方法。

（4）肺功能恢复项目

肺癌手术是损伤性手术，其所带来的肺组织缺损和肺功能下降是必然结果，手术切除范围不同，肺组织缺损和功能下降程度也不同。肺组织缺损虽无法修复，但术后合理的功能锻炼可使患者肺功能在术后尽可能短的时间内恢复至接近术前水平，其采取的主要措施包括快速康复呼吸训练操、有效咳嗽训练、吹气球训练、激励性肺活量测定法、腹式呼吸训练法、口腔卫生护理、患者与家属教育等。研究^[47⇓-49]^证实，这些术后肺功能恢复策略可缓解老年患者在术后恢复活动时的肺通气功能依赖性，有效促进血液循环，促进心肺功能早期恢复，综合提升患者的机体活动耐力，并且可以显著降低患者术后肺部并发症发生率。

## 结论

总之，老年肺癌患者外科治疗的目标是在全面评估其身体机能状态、预期寿命的基础上做出让患者获益最大的决策。通过多学科合作，进行综合术前评估，细化术中及围手术期管理，最大程度地降低手术风险，降低术后并发症发生率，缩短住院时间，提高医疗质量和效率，精准检查，使患者尽早恢复正常生活是老年肺癌患者外科治疗的最终目标。

**Table:** 

参与本共识的专家组成员	
顾问专家	
支修益	首都医科大学宣武医院
周清华	四川大学华西医院
高树庚	中国医学科学院肿瘤医院
何建行	广州医科大学附属第一医院
陈海泉	复旦大学附属肿瘤医院
胡坚	浙江大学医学院附属第一医院
执笔专家	
陈军	天津医科大学总医院
车国卫	四川大学华西医院
孙大强	天津市胸科医院
陈昶	上海市肺科医院
徐嵩	天津医科大学总医院
李昕	天津医科大学总医院
郭占林	内蒙古医科大学附属医院
付军科	西安交通大学第一附属医院
刘宏旭	大连理工大学附属肿瘤医院， 辽宁省肿瘤医院
魏煜程	青岛大学附属医院
彭忠民	山东第一医科大学附属省立医院，山东省立医院
张军航	中山大学附属第三医院
田子强	河北医科大学第四医院， 河北省肿瘤医院
张广健	西安交通大学第一附属医院
朱大兴	四川大学华西医院
朱全	江苏省人民医院， 南京医科大学第一附属医院
刘洪生	北京协和医院
茅腾	上海交通大学附属胸科医院
参与撰写专家	
陈钢	天津医科大学总医院
宋作庆	天津医科大学总医院
韦森	天津医科大学总医院
赵洪林	天津医科大学总医院
董明	天津医科大学总医院
刘京豪	天津医科大学总医院
刘明辉	天津医科大学总医院
李彤	天津医科大学总医院
任凡	天津医科大学总医院
张洪兵	天津医科大学总医院
任典	天津医科大学总医院
赵青春	天津医科大学总医院
刘仁旺	天津医科大学总医院
张子禾	天津医科大学总医院
夏春秋	天津医科大学总医院
